# Synthesis and Transformations of di*-endo*-3-Aminobicyclo-[2.2.2]oct-5-ene-2-carboxylic Acid Derivatives 

**DOI:** 10.3390/molecules16097691

**Published:** 2011-09-07

**Authors:** Márta Palkó, Pál Sohár, Ferenc Fülöp

**Affiliations:** 1 Institute of Pharmaceutical Chemistry, University of Szeged, H-6720 Szeged, Eötvös utca 6, Hungary; 2 Institute of Chemistry, Eötvös Lóránd University, H-1518 Budapest, POB 32, Hungary

**Keywords:** hydroxy-β-amino acids, cyclization, heterocycles, retro Diels-Alder reaction, microwave

## Abstract

all*-endo-*3-amino-5-hydroxybicyclo[2.2.2]octane-2-carboxylic acid (**13**) and all*-endo*-5-amino-6-(hydroxymethyl)bicyclo[2.2.2]octan-2-ol (**10**) were prepared via dihydro-1,3-oxazine or *γ*-lactone intermediates by the stereoselective functionalization of an *N*-protected derivative of *endo*-3-aminobicyclo[2.2.2]oct-5-ene-2-carboxylic acid (**2**). Ring closure of β-amino ester **4** resulted in tricyclic pyrimidinones **15** and **16**. The structures, stereochemistry and relative configurations of the synthesized compounds were determined by IR and NMR.

## 1. Introduction

The synthesis of non-natural α-amino acids is currently an important synthetic challenge in view of their increasing role in chemistry and biology. Among them, bicyclic amino acids exhibit biological activity; as an example, 2-aminobicyclo[2.2.1]heptane-2-carboxylic acid (BCH) blocks the transport of nonpolar amino acids across cell membranes, acts as an insulin-releasing factor and also inhibits the flavoprotein amino acid oxidases [1]. Straub *et al*. determined whether protein acylation plays a part in the action of glucose on insulin-secreting β-cells. They reported that BCH, a non-metabolizable analog of leucine that mimics the stimulatory effect of glucose on insulin secretion, increased the incorporation of ^3^H-palmitic acid into protein [2]. Maechler *et al*. examined the whether activation of glutamate dehydrogenase (a mitochondrial enzyme playing a key role in the control of insulin secretion) by BCH enhances glutamine oxidation and insulin secretion [3]. BHC is a model compound for the study of amino acid transporters, as it is an L-selective inhibitor that at suitable concentration can induce the suppression of cell growth and cancer cell apoptosis. [4,5] The interest in synthetic amino acids possessing a bicycle[2.2.2]octane structure is highlighted by a number of investigations relating to their biological action. Dihydroxylated derivatives of 4-aminobicyclo[2.2.2]octane-1-carboxylic acid have been used as scaffolds for antiviral agents [6,7], and 2-amino-bicyclo[2.2.2]octane-2-carboxylic acid selectively disturbs levels of neutral amino acids in the cerebral cortex [8,9]. Although of less biological importance than their α-analogs, some bicyclic β-amino acid derivatives exert biological activity [10,11], and are also present in peptides [10,12]. For example, a series of cyclic β-amino acid dipeptide derivatives have been investigated as VLA-4 antagonists in various inflammatory and autoimmune disease states [13].

During the past 20 years, a number of bicyclic β-amino acid derivatives have been synthesized, some of them with useful pharmacological effects [4], and they are widely used for the preparation of saturated 1,3-heterocycles. The synthesis and stereochemical aspects of the *diexo*- and *diendo*-fused norbornane- and norbornene-1,3-heterocycles have been thoroughly studied [14]. To date, only a few bicyclo[2.2.2]octene-fused heterocycles have been prepared [15,16,17,18,19]. Because of their therapeutic interest, the syntheses of cycloalkane-fused pyrimidinones have been studied [14], but syntheses of their bicyclo[2.2.2]octene-condensed derivatives have not yet been reported. 

*cis*- and *trans*-3-Aminobicyclo[2.2.2]octane-2-carboxylic acid were prepared some years ago [20,21,22], but their partially saturated analogs and further functionalized derivatives have not yet been described. Our work was focused on the syntheses of di*-endo*-3-aminobicyclo[2.2.2]oct-5-ene-2-carboxylic acid and its hydroxyl-substituted derivatives by stereoselective and regioselective functionalization of the double bond via 1,3-oxazine or γ-lactone intermediates. A further aim was a study of the ring-closure reactions of amino esters, and the retro-Diels-Alder reactions of the synthesized tricyclic pyrimidinones. 

## 2. Results and Discussion

**Scheme 1:**
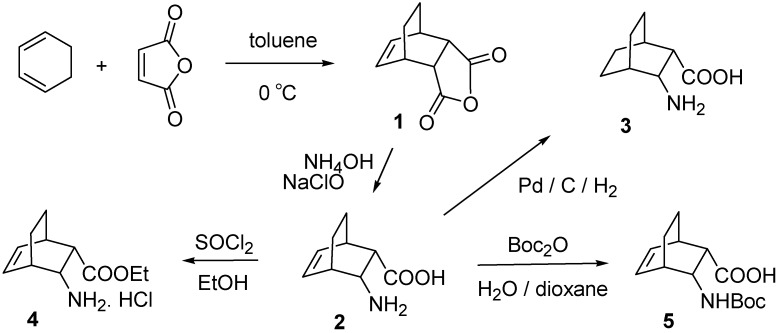
Synthesis of bicyclic amino acid derivatives **2-5**.

The Diels Alder reaction of 1,3-cyclohexadiene with maleic anhydride resulted in di*-endo*-bicyclo[2.2.2]oct-5-ene-2,3-dicarboxylic acid anhydride (**1**) diastereoseletively. The starting di*-endo*-3-aminobicyclo[2.2.2]oct-5-ene-2-carboxylic acid (**2**) was prepared selectively by hypochlorite-mediated Hoffman degradation of the carboxamide obtained by ammonolysis of anhydride **1**. Amino acid **2** was esterified in the presence of EtOH and SOCl_2_, furnishing the amino ester **4**. Compound **2** was also transformed into *cis*-amino acid **3** with H_2_ in the presence of Pd/C, and it was protected with *tert*-butoxypyrocarbonate to give *N*-acylated amino acid **5** (Scheme 1). 

We earlier reported several methods for the synthesis of β-amino acids with hydroxy-substituted cyclopentane, cyclohexane, cycloctane and norbornane skeletons. The hydroxy group could be introduced stereoselectively on the ring by starting from *cis-*, *trans-* or di*-endo*-alicyclic aminocarboxylic acids by iodolactonization or via the corresponding oxazine or oxazoline derivatives [23,24,25,26,27,28,29]. Another method of hydroxylation of 2-aminocyclohexenecarboxylic acid is feasible by functionalization of the olefinic bond through epoxidation [30].

**Scheme 2:**
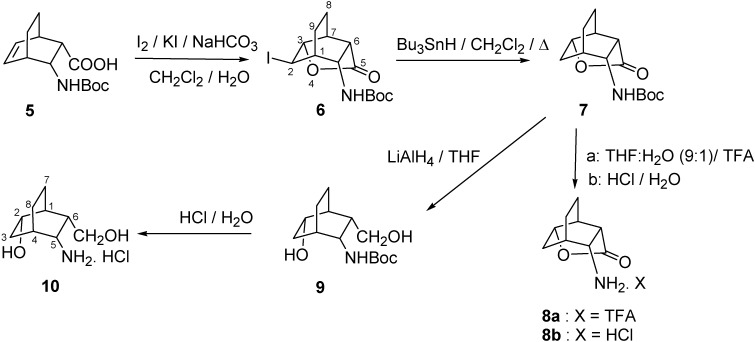
Synthesis of amino alcohol **10** via tricyclic γ-lactone intermediates.

Our present aim was the functionalization of the olefinic bond of aminocarboxylic acid derivatives **4** and **5**, and the synthesis and structural analysis of new hydroxy-substituted 3-aminobicyclo-[2.2.2]oct-5-ene-2-carboxylic acid derivatives. The first step in these syntheses was the stereoselective iodolactonization of *N*-Boc-*endo*-3-aminobicyclo[2.2.2]oct-5-ene-2-carboxylic acid (**5**) under two-phase conditions, furnishing iodolactone **6**, which was reduced with Bu_3_SnH to give *N*-Boc lactone **7**. When **7** was reacted with TFA or HCl, only the protecting group was eliminated, resulting in lactones **8a** or **8b**, instead of the all-*endo-*3-amino-6-hydroxybicyclo[2.2.2]octane-2-carboxylic acid. The similar lactone opening of **7** was also attempted with NaN_3_ [31,32,33], BF_3_.OEt_2_ [34] or LiOH [26], but not even traces of the desired product were observed in the reaction mixture. Reductive opening of the lactone ring of **7** with LiAlH_4_ in THF resulted in the protected amino alcohol **9**, and subsequent deprotection of the amino group by acidic hydrolysis afforded all-*endo*-5-amino-6-(hydroxymethyl)bicyclo[2.2.2]octan-2-ol (**10**) (Scheme 2). 

**Scheme 3:**
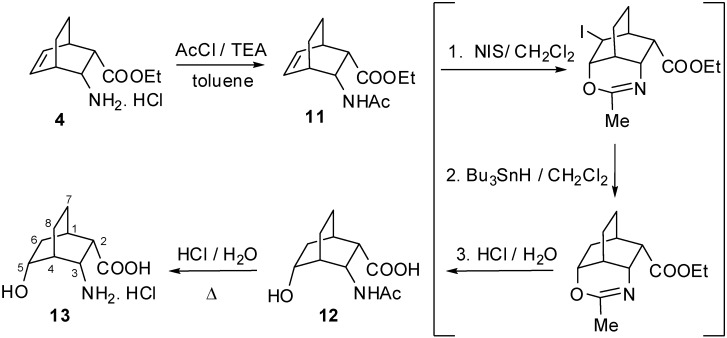
Synthesis of amino acid **13** via tricyclic 1,3-oxazine intermediates.

When *N*-acetyl derivative **11** was reacted with *N*-iodosuccinimide (NIS), a tricyclic dihydro-iodooxazine derivative was obtained regio- and stereoselectively. Not even traces of other regio- or diastereomers were observed in the crude product. Selective reduction of the halogen group with of this dihydro-iodooxazine Bu_3_SnH under an argon atmosphere led to the dihydrooxazine. Hydrolysis of this derivative with dilute HCl at room temperature gave *N*-acetylhydroxy amino acid **12**. When **12** was boiled in acidic solution, all-*endo*-3-amino-5-hydroxybicyclo[2.2.2]octane-2-carboxylic acid hydrochloride (**13**) was produced in medium yield (Scheme 3).

Amino ester base **4** reacted with PhNCS to give thiourea ester **14**. This was cyclized by acid catalysis to 5,8-ethano-3-phenyl-2-thioxo-2,3,*r*-4a,*t*-5,*t*-8,*c*-8a-hexahydroquinazolin-4(*1H*)-one (**15**). In a similar manner as for the related tricyclic 3-substituted 2-thioxo-5,8-methanoquinazolin-4-ones investigated earlier, these compounds readily underwent decomposition when heated to their melting points; cyclopentadiene was split off, and monocyclic 2,3-dihydro-2-thioxopyrimidin-4(*1H*)-ones were formed [35].

The importance of this retro Diels-Alder procedure (cycloreversion) lies in the fact that 3-substituted 2-thiouracyl derivative of type **17** can be synthesized in this way [36,37]. When **15** was boiled in chlorobenzene, or heated at the melting point, or heated under MW-irradiation, the reaction mixture turned deep-brown, but the formation of **17** was not observed, the starting thioxopyrimidinone derivative **15** was not undergone any transformation.

**Scheme 4:**
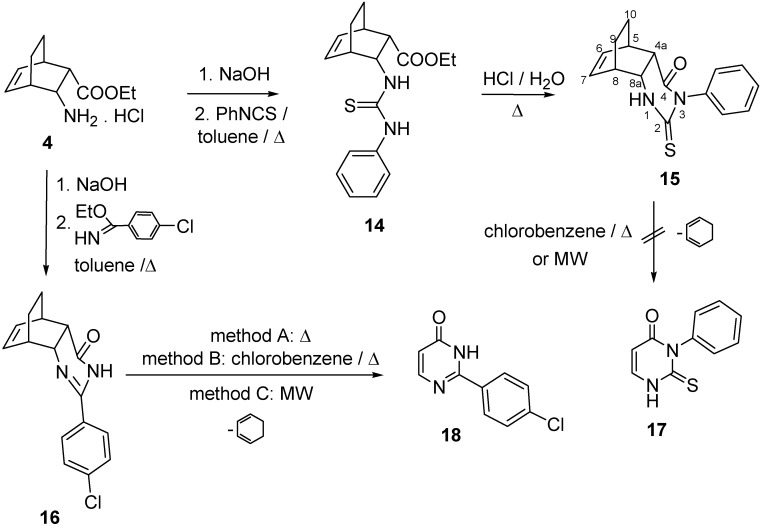
Synthesis of ethanoquinazolin-4-ones **15** and **16**, and retro Diels-Alder reaction of **16**.

When boiled in toluene with ethyl 4-chlorobenzimidate, amino ester base **4** furnished 2-(4-chlorophenyl)-5,8-ethano-*r*-4a,*t*-5,*t*-8,*c*-8a-tetrahydroquinazolin-4(*3H*)-one (**16**) in good yield. Cyclohexadiene could be split off **16** under mild conditions to give the known pyrimidin-4-(*3H*)-one **18** [37,38]. When the retro Diels-Alder reaction was carried out without any solvent, by using microwave heating, the product **18** was cleaner, the yield was higher and the reaction was faster than when **16** was boiled in chlorobenzene or heated at the melting point (Scheme 4).

### 2.1. IR and NMR Results

The presumed structures of the new compounds (**2**, **4–7**, **8a,b** and **9–16**) follow straightforwardly from the spectral data [Table 1 and Table 2; to facilitate comparison of the analogs’ spectroscopic data, the IUPAC numbering for **13** (Scheme 3) is used in this section and in Table 1 and Table 2]. The following additional remarks are necessary:

The zwitterionic structures of **2** and **3** and the ammonium salt structures of **4**, **8**, **10** and **13** are supported by the very diffuse νNH_3_^+^ band in the 3500–2000 cm^−1^ IR interval [39a]. The characteristic high νC=O frequency of **6–8** at 1762–1808 cm^−1^ is evidence of the presence of a carbonyl group in the compound the γ-lactone moiety [39b].

The presence of the 5-iodo substituent in **6** causes downfield shifts of the C–4 and C–6 lines lines in the ^13^C NMR spectrum (by 7.1 and 7.9 ppm, respectively) and an opposite change in the shift of the C–5 signal (by 4.2 ppm) as compared with **7** (β-effect), in accord with the literature [40a,^41^,^42^]. Further proof was supplied by the elemental analysis and the mass spectroscopic measurements.

The *endo* position of the 2,3-substituents is proved by the doublet split (9.5 ± 0.4 Hz) of the H−2 ^1^H NMR signal for **2–5**, **11**, **12** and **14**. The bulkier carbonyl substituent (relative to 3-NH) forces the flexible bicyclooctane skeleton into a conformation in which the dihedral angle H−1,H−2 is close to 90°, and due to the Karplus relation [42,43], the corresponding vicinal coupling is small. The mutual intensity enhancements of one of H-7*endo* and H-2 saturating the other of them (in case of compounds **4** and **5**) are unambiguous proofs of the *endo* position of the C-2 substituent. Consequently, only the ^3^*J*(H−2,H−3) interaction leads to a well-identifiable split of the H−2 signal.

**Table 1:** ^1^H NMR chemical shifts^a^ of compounds **2–7**, **8a**, **b** and **9–16** ^b^.

Compound	H–1 ^c^ ~ *s* / br	H–2 ^d^ *m* (1H)	H–3 ^e^ *m* (1H)	H–4 ^c^ ~ *s* / br	H–5 1/2 signal (1/2H) ^f^	H–6 1/2 signal (1/2H) ^f^	NH/NH_3_^+^ *br* (1/3H) ^g^	OH *br* (1H)
**2**	2.65	2.33	3.38	2.96	6.08	6.33	~8.7	–
**3**^h^	1.94	2.39	3.31	1.67	~1.5 *m* (3H), ^i^ 1.73 *t* (1H)		~8.9	–
**4**	2.80	3.2	3.56	2.96	6.16	6.33	7.95	–
**5**	2.55	2.88	4.04	2.65	6.11	6.37	5.32	12.07 ^i^
**6**	~2.2 ^j^	2.90	4.12	2.60	4.43 ~ *s* (1H)	4.96 ^k,l^	4.96 ^l^	–
**7**	2.62	2.88	3.85	1.94 ^j^	1.65, ^l^ 1.95 ^j^	4.60	4.88	–
**8a**	2.69	2.96	3.50	1.89	1.80, ^m^ 2.11 ^k^	4.75	~8.3	–
**8b**	2.64	2.84	3.42	2.04	~1.75, ^j^ 2.14 ^k^	4.71	~8.55	–
**9**^n^	~1.6 ^j^	1.88	3.75 ^l^	~1.6^ j^	1.35, ~1.65 ^j^	3.75 ^l^	5.37	4..63^o^
**10**	1.68	1.95 ^j^	3.39	1.95 ^j^	1.53, ^k^ 1.70 ^m^	3.78 ^l^	7.88	~5.1^p^
**11**	2.73	2.97	4.52	2.65	6.16	6.48	5.85	–
**12**	2.02	3.05	4.20	1.88	5.06 ^q^	1.76, ^m^ 2.23 ^q^	12.85^ r^	~3.5
**13**	2.05	2.65	3.57	2.03	3.92 ^k^	1.58,^k^ 1.89	~8.0	~5.9
**14**	2.73 ^s^	3.05	5.06	2.94 ^s^	6.05	6.37	6.81^t^	–
**15**	3.32	3.15	3.91	2.88	6.44 narrow *m* (2H) ^u^		7.75	–
**16**	3.23 ^v^	2.81	4.25	3.15 ^v^	6.25 narrow *m* (2H) ^u^		8.90	–

Further signals, CH_3_ (Et), *t* (*J*: 7.1): 1.18 (**4**,^j^ **11**^j^ and **14**^j^); CH_3_(Ac): 1.82 (**11**), 2.36 (**12**); CH_2_, (Pos. 7, 8), 1–4 *m*’s (4H): 1.0–1.9 ppm. In overlap with the H−1, H−5 or CH_3_ signal (**4**,^j^ **6**,^j^ **7**,^k^ **8b**,^j^ **9**,^j^ **11**^j^ and **14**^j^); OCH_2_, 1 or 2*m* (2H): 3.94 (**4**), ~3.52 (**9**), 3.50 and 3.78^l^ (**10**), 4.01 (**11**), 3.95 (**14**); CH_3_(Boc), *s* (9H): 1.35 (**5 **and **9**^j^), 1.42 (**6 **and **7**); Phenyl (**14**–**16**): H*^ortho^* (2H): 7.11, 7.05 *br* and 7.15 *br*, 7.69, H*^meta^* (2H): 7.38, ~7.4,^j^ 7.40, H*^para^* (1H): 7.25, ~7.4.^j^ ^a^ In ppm (δ_TMS_ = 0 ppm) at 125.7 MHz. Solvent: DMSO-d_6_; for **6**, **7**, **11** and **14–16**: CDCl_3_; ^b^ Assignments were supported by 2D-HMQC (except for **3** and **7**), 2D-HMBC (except for **3**, **7**, **9 **and **10**), 2D-COSY (**9 **and **10**) and DIFFNOE measurements (**4**, **5**, **8b** and **16**); ^c^ Singlet-like or broad signal (1H) with close-lying coalesced lines; ^d^ *d* (1H), *J*: 9.1 (**2**, **4**), 9.6 (**3**, **11**, **12 **and **14**), 9.8 (**5**), 14.5 (**10**), 5.8 (**13**), *dd* (1H), *J*: 9.5 and 4.8 (**6**, **7**, **8a** and **8b**), 10.4 and 2.5 (**15** and **16**), *m* (1H, **9**); ^e^ Multiplicity and *J*-values are the same as for H−2 (**2**, **3**, **13**, **16**), in case of **4**, **5**, **11**, **12**, **14** and **15** further split by 2.5±0.5 Hz, the *dd* of H−2 is coalesced to a ~*s* (**6**), ~*t* (**7**) or *d* (**8a**,**b**); ^f^ *t* (1H), *J*: 7.3 (**2**, **4**, **5**, **11 **and **14**), 6.2 (for H−6 of **7** and **8a**,**b**), 11.0 (for the H−6 *t* of **13 **at 1.89); ^g^ NH_3_^+^ (3H) for **2–4**, **8a,b**, **10** and **13**. NH, *d*(1H), *J*: 10 (**5**, **11**), broad, 1H (**6**, **7 **and **14–16**), 3.1 (**9**), separated signal of COOH at 12.7 ppm (**13**); ^h^ Known [20], zwitterionic molecule; ^I^ COOH; ^j, l^ Overlapping signals; ^k^ *d* (1H), *J*: 5.1 (**6**), 14.5 (**10**), 7.7 (**13**, H−5), 13.5 (**13,** H−6), ~*d* with coalesced lines (**8a**,**b**); ^m^ *dd* (1H) with coalesced lines (**8a**, **10 **and **12**); ^n^ Contaminated with 10-15% 5,6-unsaturated analog; ^o^ *t* (*J*: 5.3), OH (Pos. 6): 6.23 *d*(*J*: 8.8); ^p^ *~s* (1H), OH (Pos. 6): 5.70 *~s*; ^q^ *m* (1H); ^r^ Coalesced with the COOH signal, broad (2H); ^s^ Reversed assignment is also possible; ^t^ NH attached to C−3 of the bicycle. NH(Ph): 8.01 br (1H); ^u^ AB spectrum with close-lying lines; ^v^ The assignment was proved by DIFFNOE measurement.

**Table 2:** ^13^C-NMR chemical shifts^a^ of compounds **2–7, 8a,b** and **9–16** ^a,b^.

Compound	C–1	C–2	C–3	C–4	C–5	C–6	C–7	C–8	C=O
**2**	36.0	46.3	50.8	35.0	130.3	137.4	25.7	23.6	175.6
**3**^c^	29.2 ^d^	43.5	48.9	29.1 ^d^	25.2 ^e^	25.9 ^e^	19.2	21.9	176.3
**4**	32.92 ^d^	46.0	51.4	32.96 ^d^	130.8	135.8	24.7	22.5	171.8
**5**	36.2	50.2	52.1	33.2	130.6	136.5	25.3	22.7	174.6
**6**	39.1	42.5	48.1	37.4	25.8	86.2	14.9	25.0	176.2
**7**	37.1	43.9	48.1	30.3	30.0	78.3	15.6	26.4	177.6
**8a**	37.1	42.4	48.4	28.6	29.3	78.6	15.1	26.3	177.0
**8b**	37.2	42.3	48.4	28.3	29.3	78.5	15.2	26.4	176.9
**9^f^**	34.3 ^d^	41.2	49.8	30.5 ^d^	31.8	68.0	25.2	23.4	−
**10**	33.8	40.4	50.1	28.4	30.6	67.3	24.1	22.9	−
**11**	33.3	49.9	50.3	35.9	130.4	136.6	25.4	22.2	173.5
**12**	25.5 ^d^	46.7	45.4	23.0 ^d^	76.8	32.3	24.2	17.5	172.6 ^e^
**13**	33.3	47.8	49.8	28.9	67.5	38.0	19.7	21.5	174.6
**14**	33.9 ^d^	49.2	57.0	35.3 ^d^	130.5	136.5	25.5	22.1	173.4
**15**	35.6	43.5	54.6	37.5	133.4	134.1	24.2	22.0	167.7
**16**	34.4 ^d^	44.3	61.6	37.5 ^d^	135.0	133.4	25.3	23.4	172.6

Further signals, CH_3_ (ethyl group): 14.8 (**4**), 14.5 (**11**, **14**); CH_3_(Ac): 23.8 (**11**); OCH_2_: 61.4 (**4**), 62.7 (**9**), 60.8 (**11**), 61.1 (**14**); CH_3_(BOC): 29.0 (**5** and **9**), 28.7 (**6 **and **7**); C*_quat_* (Boc): 78.9 (**5**), 80.8 (**6**), 80.3 (**7**), 78.5 (**9**); C=O (Boc): 155.3 (**5**), 155.8 (**6 **and **7**), 156.1 (**9**); C=O (amide): 169.3 (**11**), 172.5 ^e^ (**12**); phenyl, C*_subst._* (**14**–**16**): 136.3, 139.1, 132.8, C*_ortho_*: 125.1, ? (broad), 128.1, C*_meta_*: 130.3, 128.9, 129.3, C*_para_*: 127.4, 129.3, 137.3; C=S: 180.6 (**15**); C=N: 146.6 (**16**). ^a^ In ppm (δ_TMS_ = 0 ppm) at 125.7 MHz. Solvent: DMSO-d_6_; for **6**, **7**, **11** and **14**–**16**: CDCl_3_; ^b^ Assignments were supported by 2D-HMQC (except for **3** and **7**), 2D-HMBC (except for **3**, **7**, **9 **and **10**) and DEPT (except for **7** and **8a**); ^c^ Known compound [20]; ^d,e^ Interchangeable assignments; ^f^ Contaminated with 10–15% 5,6-unsaturated analog.

In **6–8**, the condensed γ-lactone ring forces the molecules into a stereo structure in which the dihedral angle H−1,H−2 is smaller, while the angle H−2,H−3 remains practically unaltered. Thus, both interactions lead to well-observable splits and the H−2 signal appears as a double doublet.

The zwitterionic and strained (condensed γ-lactone ring) structures of **2**, **3** and **6–8**, are manifested, as expected [40b], in low field shifts of the C=O line (175.6, 176.3 and 176.9 ± 0.7 ppm, respectively) relative to the values measured for the other compounds (171.8–174.6 ppm) (thioimide **15** is an exception for which this line is at 167.7 ppm, in accord with the literature data [40b]) in the diendo-position. 

In **10**, the steric interaction between the 2-hydroxymethyl and 6-hydroxy groups in the di*-exo-*position compensates the effort of the bulkier NH_3_^+^ group to occupy an out of plane position (relative to the plane of the methylene carbon and C-2,3), and consequently the cyclohexane ring bearing three substituents is forced into a nearly ideal boat conformation (in contrast with the other compounds discussed above), with a dihedral angle H−2,H−3 of ca. 0°. Thus, this compound exhibits the highest split (14.5 Hz) of the H−2 doublet.

As a result of steric hindrance of the substituents in **13**, here in Pos. 2, 3 and 5, the dihedral angle H−2,H−3 is most distant from 0° and the corresponding split is the smallest (5.8 Hz).

In pyrimidone-condensed **15** and **16**, the anisotropy of the neighbouring carbonyl [40c] results in a downfield shift of the H−1 signal (3.32 and 3.23 ppm), in contrast with the values of 1.68–2.80 ppm measured for the other compounds.

The strained skeleton in **6–8** and the steric hindrance in **9** and **10** (between the *diendo* substituents in Pos. 2 and 6) show up in upfield shifts (steric compression shifts or field effects [40d]) of the C−2 line (at 40.4–43.9 ppm) as compared with the values observed for **2**, **4**, **5** and **11–14** (46.0–50.2 ppm). In **15** and 1**6**, a similar situation due to the condensed heteroring also leads to strain in the molecular skeleton as proved by upfield shifts of involved carbon signals.

Similarly, the C−7 line is upfield-shifted (14.9–15.6 ppm) for **6**, **7** and **8a,b**. In the other cases, these shifts are between 24.1 and 25.7 ppm (except for **3** and **13**, where the bulky NH_3_^+^ and the 7-CH_2_ groups are also in steric interaction (19.2 and 19.7 ppm)).

Mention should be made of the significant downfield shift of the *exo* H−8 signal in **6** (at 2.22 ppm, whereas in **7** this shift is ca. 1.75 ppm), which originates from the anisotropic effect of the iodo substituent [40e] at Pos. 5. 

## 3. Experimental

### 3.1. General

The chemicals were purchased from Aldrich or Fluka. Melting points were determined on a Kofler micro melting point apparatus. Elemental analyses were performed with a Perkin-Elmer CHNS-2400 Ser II Elemental Analyser; Merck Kieselgel 60F_254_ plates were used for TLC: the eluent was 4:1 toluene-MeOH. Products were purified by column chromatography on Merck 0.063–0.2 mm silica gel; the elution mixtures were determined case by case. Microwave reactions were performed in a CEM Discover LabMate MW reactor. The ^1^H- and ^13^C-NMR spectra were recorded in CDCl_3_ or DMSO-d_6_ solution in 5 mm tubes at room temperature, on a Bruker DRX 500 spectrometer at 500 (^1^H) and 125 (^13^C) MHz, with the deuterium signal of the solvent as the lock and TMS as internal standard. The standard Bruker microprogram NOEMULT.AU to generate NOE was used. DEPT spectra were run in a standard manner, using only the = 135° pulse to separate CH/CH_3_ and CH_2_ lines phased “up” and “down”, respectively. The 2D-HSC spectra were obtained by using the standard Bruker pulse program HXCO.AU.

*di-endo-3-Aminobicyclo[2.2.2]oct-5-ene-2-carboxylic acid* (**2**): di*-endo-*Bicyclo[2.2.2]oct-5-ene-2,3-dicarboxylic acid anhydride (**1**, 6,4 g, 30 mmol) was added in portions to dilute NH_4_OH (50 mL, 6%) at 0 °C. The mixture was stirred for 30 min, and 2 M NaOH (60 mL) was then added dropwise at 0 °C over a period of 30 min, after which the excess of NH_3_ was removed under reduced pressure at 40 °C. The residue was cooled to 0 °C and 1 M NaClO solution (40 mL) was added dropwise with stirring, the temperature being maintained at 0 °C throughout. The mixture was stirred at the same temperature for 1 h, held at 70–75 °C for 10 min, then cooled to ambient temperature, adjusted with 10 M HCl to pH 7 and evaporated to dryness. The residue was extracted with three 150 mL portions of hot MeOH, and the extract was evaporated. The residue was dissolved in a small amount of water and the HCl was removed by means of a Dowex 50 ion-exchange column (acid cycle). Elution was effected with 1 M NH_4_OH solution. Each fraction was evaporated and the dry residue was dissolved in water, acetone was added until turbidity appeared, and the mixture was then allowed to stand in a refrigerator. The solid crystals were filtered off. Yield 3.35 g (66%); m.p. 204–208 °C C_9_H_13_NO_2_ (167.09): calcd. C 64.65, H 7.84; N 8.38, found C 64.77, H 7.96, N 8.43.

*cis-3-Aminobicyclo[2.2.2]octane-2-carboxylic acid* (**3**): A solution of amino acid **2** (350 mg, 2.1 mmol) and 10% Pd/C (100 mg) in MeOH (100 mL) was stirred under H_2_ (50 atm) for 3 days at room temperature. The Pd was then filtered off and the filtrate was concentrated under reduced pressure. The residue was crystallized from water-acetone. Yield 0.18 g (51%); a white solid, m.p. 215–220 °C, lit. m.p. 232–235 °C (HCl salt) [20] C_9_H_15_NO_2_ (169.11): calcd. C 63.88, H 8.93; N 8.28, found C 64.02, H 8.12, N 8.19.

*Ethyl di-endo-3-aminobicyclo[2.2.2]oct-5-ene-2-carboxylate* (**4**): SOCl_2_ (2.6 mL, 35 mmol) was added dropwise with stirring to absolute EtOH (30 mL) at −10 °C. *cis*-3-Aminobicyclo[2.2.2]octane-2-carboxylic acid (**3**, 5.5 g, 33 mmol) was added in portions to the mixture, which was stirred for 30 min at 0 °C, and then for 3 h at room temperature, after which the mixture was refluxed for 1 h and next evaporated. The residue was crystallized from Et_2_O and recrystallized from EtOH/ Et_2_O). Yield 6.6 g (87%); a white solid, m.p. 209–213 °C, C_11_H_18_ClNO_2_ (231.10): calcd. C 57.02, H 7.83; Cl: 15.30, N 6.04, found C 57.22, H 7.92, Cl, 15.38, N 6.19.

*di-endo-3-tert-Butoxycarbonylaminobicyclo[2.2.2]oct-5-ene-2-carboxylic acid* (**5**): 1 M NaOH (20 mL) was added to a solution of 3-aminobicyclo[2.2.2]oct-5-ene-2-carboxylic acid (**2**, 3.34 g, 20 mmol) in a 2:1 dioxane/H_2_O mixture (60 mL). The solution was cooled to 0 °C in an ice bath and di-*tert*-butyl dicarbonate (4.8 g, 22 mmol) was added slowly. The mixture was stirred at 0 °C for 30 min and then warmed to room temperature and stirred for 4 h. The solvent was concentrated to 20 mL, the pH was then adjusted to 2.5 with 10% H_2_SO_4_, and the resulting solution was extracted with EtOAc (3 × 50 mL). The combined extracts were dried (Na_2_SO_4_) and evaporated, to give **5** as a white solid, which was recrystallized from *i*Pr_2_O. Yield 3.4 g (63%); m.p. 117–120 °C C_14_H_21_NO_4_ (267.15): calcd. C 62.90, H 7.92, N 5.24, found C 62.78, H 7.99, N 5.11.

*(r-1,c-2,t-3,t-6,c-7,t-10)-10-tert-Butoxycarbonylamino-2-iodo-4-oxatricyclo[4.3.1.0^3,7^]decan-5-one* (**6**): To a solution of **5** (3.04 g, 11.4 mmol) in CH_2_Cl_2_ (100 mL), NaHCO_3_ solution (0.5 M, 70 mL), KI (11.62 g, 70 mmol) and I_2_ (5.84 g, 23 mmol) were added at 0 °C. The reaction mixture was stirred at room temperature for 20 h and then poured into 10% aqueous Na_2_S_2_O_3_ solution (50 mL). The reaction mixture was extracted with 3 × 20 mL CH_2_Cl_2_ and the combined extract was washed with brine (20 mL), dried (Na_2_SO_4_) and evaporated. The residue was recrystallized from *i*Pr_2_O. Yield 2.9 g (64%); m.p. 172–174 °C. C_14_H_20_INO_4_ (393.04): calcd. C 42.76, H 5.13, N 3.56, found C 42.91, H 5.09, N 3.61.

*(r-1,t-3,t-6,c-7,t-10)-10-tert-Butoxycarbonylamino-4-oxatricyclo[4.3.1.0^3,7^]decan-5-one* (**7**): Bu_3_SnH (4.8 mL, 18 mmol) was added to a solution of iodolactone **6** (3.53 g, 9 mmol) in dry CH_2_Cl_2_ (65 mL) under Ar. After stirring at 40 °C for 20 h, the solvent was evaporated off, and the residue was crystallized from *n*-hexane and recrystallized from *i*Pr_2_O-EtOAc. Yield 1.99 g (83%); m.p. 172–174 °C C_14_H_21_NO_4_ (267.15): calcd. C 62.90, H 7.92, N 5.24, found C 62.81, H 8.08, N 5.31.

### 3.2. (r-1,t-3,t-6,c-7,t-10)-10-Amino-4-oxatricyclo[4.3.1.0^3,7^]decan-5-one trifluoroacetate *(**8a**)* and hydrochloride *(**8b**)*

**8a**: Trifluoroacetic acid (20 mL) was added to a solution of Boc-lactone derivative **7** (0.35 g, 13 mmol) in a 9:1 THF:H_2_O mixture (60 mL) and the solution was stirred at room temperature for 10 h. The solvent was next evaporated off and the residue was crystallized from Et_2_O and recrystallized from H_2_O-acetone. Yield 0.19 g (54%); m.p. 235–236 °C C_11_H_13_F_3_NO_3_ (264.08): calcd. C 50.00, H 4.96, N 5.30, found C 50.11, H 5.13, N 5.41.

**8b**: Compound **7** (0.4 g, 2 mmol) was dissolved in aqueous HCl (20%, 20 mL) and the solution was stirred at room temperature for 10 h. The solvent was next evaporated off and the residue was recrystallized from H_2_O-acetone. Yield 0.2 g (75%); m.p. 256–260 °C. C_9_H_14_ClNO_2_ (203.07): calcd. C 53.08, H 6.93, Cl: 17.41, N 6.88, found C 53.21, H 6.98, Cl: 17.54, N 6.61.

*all-endo-tert-Butyl-N-[5-(hydroxy-3-hydroxymethyl)bicyclo[2.2.2]octan-2-yl] carbamate* (**9**): To a stirred suspension of LiAlH_4_ (1 g, 26 mmol) in dry THF (60 mL) was added a solution of Boc-lactone **7** (0.5 g, 1.9 mmol) in dry THF (20 mL). The resulting suspension was refluxed for 4 h and then decomposed by the addition of a mixture of water (2 mL) and THF (10 mL). The inorganic material was filtered off and washed with THF (3 × 50 mL). After drying (Na_2_SO_4_) and filtration, the solvent was evaporated off to give a pale oil, which was purified by column chromatography (toluene-MeOH = 4:1) Yield 0.41 g (81%) C_14_H_25_NO_4_ (271.18): calcd. C 61.97, H 9.29, N 5.16, found C 62.08, H 9.41, N 5.31.

*all-endo-5-Amino-6-(hydroxymethyl)bicyclo[2.2.2]octan-2-ol hydrochloride* (**10**): Compound **9** (0.4 g, 2 mmol) was dissolved in aqueous HCl (20%, 20 mL) and the solution was stirred at room temperature for 1 h. The solvent was next evaporated off and the residue was recrystallized from H_2_O-acetone. Yield 0.3 g, (72%); m.p. 165–167 °C. C_9_H_18_ClNO_2_ (207.10): calcd. C 52.05, H 8.74, Cl: 17.07, N 6.74, found C 52.24, H 8.92, Cl: 17.24, N 6.68.

*Ethyl di-endo-3-acetylaminobicyclo[2.2.2]oct-5-ene-2-carboxylate* (**11**): To a suspension of ethyl 3-aminobicyclo[2.2.2]oct-5-ene-2-carboxylate hydrochloride (**4**, 3 g, 13 mmol) in CHCl3 (50 mL), Et_3_N (3.8 mL, 26 mmol), and AcCl (1.1 mL, 15 mmol) were added and the reaction mixture was stirred at room temperature for 2 h, and then washed with H_2_O (2 × 20 mL). The aqueous layer was extracted with EtOAc (3 × 30 mL). The combined organic layer was dried (Na_2_SO_4_) and evaporated. The residue was recrystallized from *n*-hexane-*i*Pr_2_O. Yield 2.42 g (78%); m.p. 120–122 °C. C_13_H_19_NO_3_ (237.14): calcd. C 65.80, H 8.07, N 5.90, found C 65.94, H 8.32, N 5.78.

*all-endo-3-Acetylamino-5-hydroxybicyclo[2.2.2]octane-2-carboxylic acid* (**12**): A solution of **11** (2.42 g, 10.21 mmol) in CH_2_Cl_2_ (80 mL) was treated with NIS (2.3 g, 10.21 mmol) and subsequently stirred for 14 h at room temperature. When the reaction was completed, the mixture was washed with 10% NaOH solution (3 × 10 mL). The aqueous solution was extracted with CH_2_Cl_2_ (3 × 40 mL) and the organic phase was dried (Na_2_SO_4_) and evaporated. The oily dihydroiodooxazine product was sensitive to air and it was therefore used without purification in the next step.

Bu_3_SnH (4 mL) was added to a solution of oily dihydroiodooxazine (2.5 g) in dry CH_2_Cl_2_ (65 mL) under Ar. After stirring for 20 h at 40 °C, the solvent was evaporated off and the residue was purified by column chromatography on silica gel (*n*-hexane:EtOAc 10:1) to afford the dihydrooxazine derivative as a colorless oil (1.05 g, 64%). This oily product was also sensitive to air; it was therefore used immediately. A solution of oily dihydrooxazine derivative (1.05 g) in 20% aqueous HCl (20 mL) was stirred for 2 h. The solvent was then evaporated off to afford crude **12**, which was recrystallized from H_2_O-acetone. Total yield 0.75 g (33%); m.p. 211–218 °C (with decomposition) C_11_H_17_NO_4_ (227.12): calcd. C 58.14, H 7.54, N 6.16, found C 58.26, H 7.72, N 6.32.

*all-endo-3-Amino-5-hydroxybicyclo[2.2.2]octane-2-carboxylic acid hydrochloride* (**13**): A solution of 0.75 g (3.3 mmol) **12** in 20% aq. HCl (30 mL) was refluxed for 30 h. The solvent was then evaporated off to afford crude **9**, which was recrystallized from EtOH-Et_2_O. Yield 0.5 g (67%); m.p. 222–230 °C (with decomposition) C_9_H_16_ClNO_3_ (221.08): calcd. C 48.76, H 7.27, Cl: 15.99, N 6.32, found C 48.64, H 7.12, Cl 16.14, N 6.38.

*Ethyl di-endo-3-phenylthiocarbamoylbicyclo[2.2.2]octane-2-carboxylate* (**14**): To a magnetically stirred toluene solution of amino ester base **4** (0.7 g, 3.6 mmol in 20 mL), one equivalent of PhNCS in toluene (0.5 g, 20 mL) was added dropwise [the free base was obtained from the hydrochloride **4** by treatment with aqueous NaOH and extraction with CHCl_3_, followed by drying (Na_2_SO_4_) and evaporation]. The mixture was refluxed for 10 h, the reaction mixture was then evaporated and the oily product was crystallized from *n*-hexane and recrystallized from *i*Pr_2_O-EtOAc. Yield 0.68 g (57%); m.p. 110–112 °C. C_18_H_22_N_2_O_2_S (330.14): calcd. C 65.42, H 6.71, N 8.48 found C 65.61, H 6.59, N 8.58.

*(r-4a,t-5,t-8,c-8a)-5,8-Ethano-3-phenyl-2-thioxo-2,3,4a,5,8,8a-hexahidroquinazolin-4(1H)-one* (**15**): The thiocarbamoyl compound **14** (2.5 mmol) was refluxed in 20% aqueous HCl (30 mL) for 3 h. The reaction mixture was then evaporated, Et_2_O was added, and the crystalline product **15** was filtered off and recrystallized from *i*Pr_2_O-EtOAc. Yield 0.68 g (57%); m.p. 285–289 °C. C_16_H_16_N_2_OS (284.10): calcd. C 67.58, H 5.67, N 9.85, found C 67.61, H 5.79, N 9.53.

*(r-4a,t-5,t-8,c-8a)-2-(4-Chlorophenyl)-5,8-ethano-4a,5,8,8a-tetrahydroquinazolin-4(3H)-one* (**16**): To a magnetically stirred toluene solution of amino ester base **4** (0.7 g, 3.6 mmol in 20 mL), one equivalent of ethyl *p*-chlorobenzimidate in toluene (0.7 g, 20 mL) and a catalytic amount of *p*-toluene-sulfonic acid was added [the free base was obtained from the hydrochloride **4** by treatment with aqueous NaOH and extraction with CHCl_3_, followed by drying (Na_2_SO_4_) and evaporation]. The mixture was refluxed for 12 h, the reaction mixture was next evaporated and the residue was recrystallized from EtOH. Yield 0.65 g (63%); m.p. 210–215 °C. C_16_H_15_ClN_2_O (286.09): calcd. C 67.02, H 5.27, C: 12.36, N 9.77, found C 67.21, H 5.58, Cl: 12.48, N 9.68.

### 3.3. 2-(4-Chlorophenyl)-3H-pyrimidin-4-one *(**18**)*

*Method A*: (*r*-4a,*t*-5,*t*-8,*c*-8a)-2-(4-Chlorophenyl)-5,8-ethano-4a,5,8,8a-tetrahydroquinazolin-4(*3H*)-one (**16**, 0.28 g, 1 mmol) was heated in a round-bottomed flask for 30 min at 220 °C. After the mixture had cooled, the residue was recrystallized from EtOH. Yield 0.12 g (58%).

*Method B*: (*r*-4a,*t*-5,*t*-8,*c*-8a)-2-(4-Chlorophenyl)-5,8-ethano-4a,5,8,8a-tetrahydroquinazolin-4(*3H*)-one (**16**, 0.28 g, 1 mmol) was refluxed in chlorobenzene (20 mL) for 12 h. The mixture was evaporated, and the residue was recrystallized from EtOH. Yield 0.13 g (63%). 

*Method C*: (*r*-4a,*t*-5,*t*-8,*c*-8a)-2-(4-Chlorophenyl)-5,8-ethano-4a,5,8,8a-tetrahydroquinazolin-4(*3H*)-one (**16**, 0.28 g, 1 mmol) was weighed into a 10 mL pressurized reaction vial and the crystals were heated at 250 °C for 5 min at max. 300 W microwave irradiation. The crude product was recrystallized from EtOH. Yield 0.15 g (72%) m.p. 243–245 °C, lit. m.p. 245–246 °C, [38]. C_10_H_7_ClN_2_O (206.02): calcd. C 58.13, H 3.41, Cl 17.16, N 13.56, found C 58.31, H 3.59, Cl 17.34, N 13.68.

## 4. Conclusions

In summary, we have successfully synthetized di*-endo*-3-aminobicyclo[2.2.2]oct-5-ene-2-carboxylic acid derivatives, can be used for further valuable transformations, and are good starting materials for which the syntheses of hydroxy-substituted β-amino acids, aminodiols and heterocycles with potential biological activity.
